# Protein expression patterns and metal metabolites in a protogynous hermaphrodite fish, the ricefield eel (*Monopterus albus*)

**DOI:** 10.1186/s12864-024-10397-w

**Published:** 2024-05-21

**Authors:** Zhi He, Feng Xiao, Deying Yang, Faqiang Deng, Wenxiang Ding, Zhide He, Siqi Wang, Qiqi Chen, Haochen Wang, Mingqiang Chen, Kuo Gao, Jinxing Xiong, Ziting Tang, Mingwang Zhang, Taiming Yan

**Affiliations:** 1 College of Animal Science and Technology, Sichuan Agricultural University, Chengdu, 611130 China; 2 Fish Resources and Environment in Upper Reaches of the Yangtze River Observation and Research Station of Sichuan Province, College of Animal Science and Technology, Sichuan Agricultural University, Chengdu, 611130 China

**Keywords:** *Monopterus albus*, Proteomic analysis, Sex change, Metal metabolites

## Abstract

**Background:**

The ricefield eel *Monopterus albus* undergoes a natural sex change from female to male during its life cycle, and previous studies have shown the potential mechanisms of this transition at the transcriptional and protein levels. However, the changes in protein levels have not been fully explored, especially in the intersexual stage.

**Results:**

In the present study, the protein expression patterns in the gonadal tissues from five different periods, the ovary (OV), early intersexual stage gonad (IE), middle intersexual stage gonad (IM), late intersexual stage gonad (IL), and testis (TE), were determined by untargeted proteomics sequencing. A total of 5125 proteins and 394 differentially expressed proteins (DEPs) were detected in the gonadal tissues. Of the 394 DEPs, there were 136 between the OV and IE groups, 20 between the IM and IE groups, 179 between the IL and IM groups, and 59 between the TE and IL groups. Three candidate proteins, insulin-like growth factor 2 mRNA-binding protein 3 isoform X1 (Igf2bp3), triosephosphate isomerase (Tpi), and Cu-Zn superoxide dismutase isoform X1 [(Cu-Zn) Sod1], were validated by western blotting to verify the reliability of the data. Furthermore, metal metabolite-related proteins were enriched in the IL vs. IM groups and TE vs. IL groups, which had close relationships with sex change, including Cu^2+^-, Ca^2+^-, Zn^2+^- and Fe^2+^/Fe^3+^-related proteins. Analysis of the combined transcriptome data revealed consistent protein/mRNA expression trends for two metal metabolite-related proteins/genes [*LOC109953912* and *calcium Binding Protein 39 Like* (*cab39l*)]. Notably, we detected significantly higher levels of Cu^2+^ during the sex change process, suggesting that Cu^2+^ is a male-related metal metabolite that may have an important function in male reproductive development.

**Conclusions:**

In summary, we analyzed the protein profiles of ricefield eel gonadal tissues in five sexual stages (OV, IE, IM, IL, and TE) and verified the plausibility of the data. After preforming the functional enrichment of metal metabolite-related DEPs, we detected the contents of the metal metabolites Zn^2+^, Cu^2+^, Ca^2+^, and Fe^2+^/Fe^3+^ at these five stages and screened for (Cu-Zn) Sod1 and Mmp-9 as possible key proteins in the sex reversal process.

**Supplementary Information:**

The online version contains supplementary material available at 10.1186/s12864-024-10397-w.

## Background

Sex determination exhibits significant diversity and variability among animals, where it is often thought to be driven by so-called master sex-determining genes or multiple environmental factors [1–4]. Some fish have a long history of undergoing sex change, the process of transitioning from one sex to another [5–7]. Thus, hermaphroditic fish provide an interesting model for the study of the mechanisms of sex determination and differentiation in vertebrates. There are three kinds of hermaphroditic fish: protogynous (female-to-male sex change), such as the ricefield eel *Monopterus albus* [8], *Lutjanus campechanus* [9], wrasse (blue-headed wrasse *Thalassoma bifasciatum* and New Zealand spotted wrasse *Notolabrus celidotus* [10]; protandry (male-to-female sex change), e.g., *Amphiprion bicinctus* [11] and the black sea bream (*Acanthopagrus schlegelii*) [12]; and bidirectional, such as two genera of small gobies (*Gobiodon* and *Paragobiodon*) [13] and the chalk bass *Serranus tortugarum* [14].

The ricefield eel is an economically important fish in China and Southeast Asia and is also a good model species for studying sex change, as they naturally change from female to male [15]. After reaching sexual maturity, the ricefield eel is a female first, and after laying eggs, the ovary gradually changes into a testis. The sex change process of the ricefield eel includes five periods: ovary (OV), early intersexual stage gonad (IE), middle intersexual stage gonad (IM), late intersexual stage gonad (IL), and testis (TE) [16–18]. Based on the transcriptome data, a few genes involved in the regulation of the sex change in the ricefield eel, such as *transforming growth factor beta 3* (*tgfb3*), *follicle stimulating hormone receptor* (*fshr*), *calcium/calmodulin dependent protein kinase 4* (*camk4*) and *calmodulin* may be the key genes, which helps balance germ cell apoptosis and proliferation during sex change [19]. Most differentially expressed genes (DEGs; mRNAs, circRNAs, and lncRNAs) were primarily enriched in the intersexual stages (IE, IL, and IM) [19–21]. The DEGs are involved in endocytosis, autophagy, and p53 apoptosis signaling [19]. In addition, two-dimensional electrophoresis was used to analyze differentially expressed proteins (DEPs) during the three phases of sex change in the ricefield eel, including the ovary, ovotestis and testis [22]. DEPs in the ovotestis phase have two functions related to ovarian apoptosis and testicular differentiation [22]. Sex change is a complex biological process [3, 6]. Although the process of sex change are now described for involving in the behavioral, gonadal, and morphological modifications, the genetic cascade orchestrating this transformation needs deeper exploration [23]. At the transcriptional level, many genes were differentially expressed during the sex change in ricefield eel, especially during the IE, IM, and IL stages, such as *forkhead box L2* (*foxl2*) and *wnt family member 4* (*wnt4*) expression was high in OV and IE gonad, and *SRY-box transcription factor 9* (*sox9*), *doublesex and mab-3 related transcription factor 1* (*dmrt1*), *dmrt2* and *dmrt3* in the TE stage were more higher than those in the OV and intersexual stages [19]. The determination of the proteome in the previous study could not completely reveal the protein patterns during the sex change process [22].

To date, the biological process of sex change is not very clear. Therefore, it is necessary to comprehensively uncover the protein expression profiles during the sex change period. In our study, the protein profiles in gonadal tissues of ricefield eel in five sexual stages (OV, IE, IM, IL, and TE) were determined and quantified. Then, the functional enrichment of DEPs was analyzed, and the metal metabolites-related proteins were identified. Furthermore, western blotting was utilized to detect the expression levels of the DEPs. The contents of metal metabolites (Zn^2+^, Cu^2+^, Ca^2+^, and Fe^2+^/Fe^3+^) were detected in tissues in these five sexual stages. The results here lay a foundation for the study of functional proteins during the process of sex change in ricefield eel and further provide references for the mechanism of sex change and fish hermaphroditism.

## Results

### Protein profiles during sex change

**Fig. 1:**
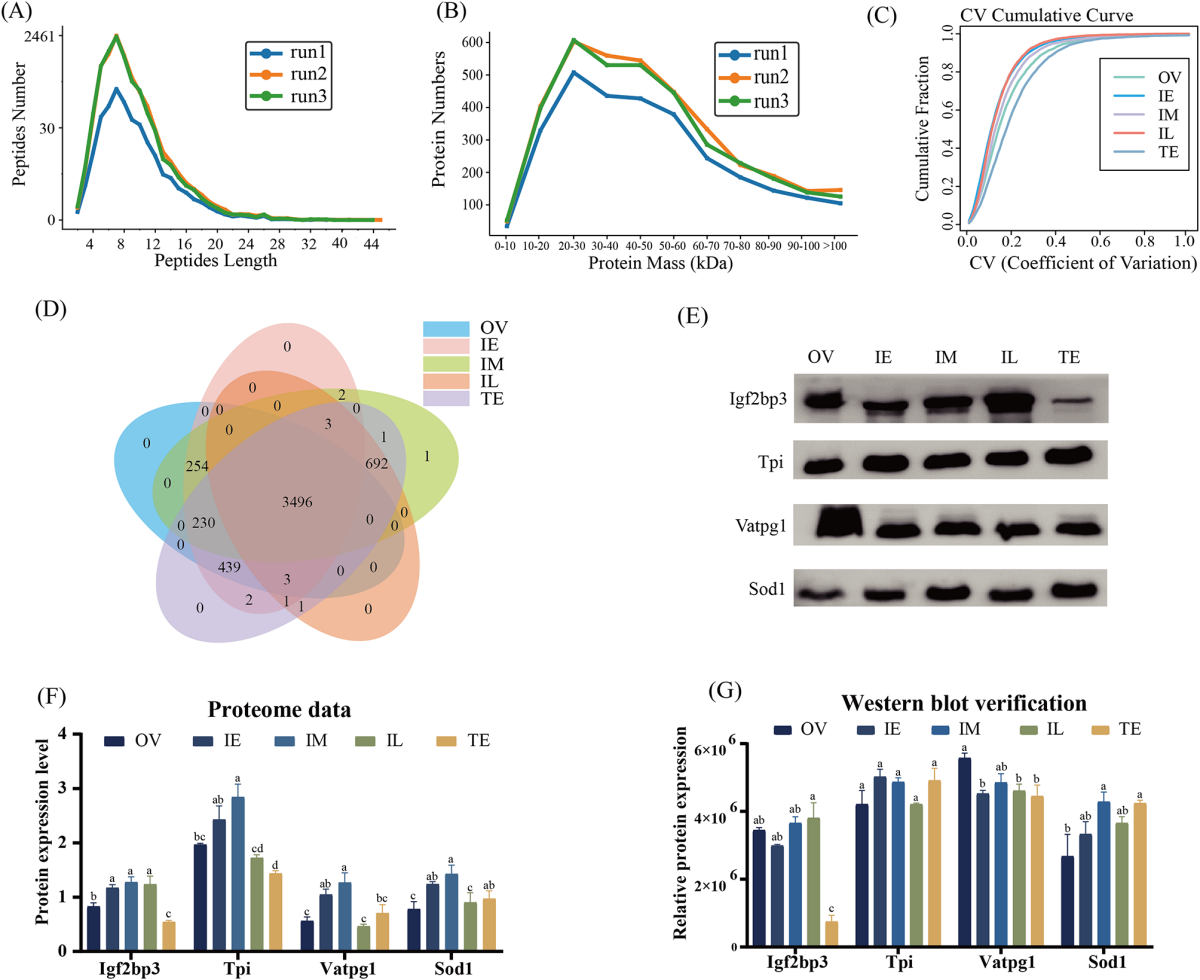
Characterization and identification of total proteins and western blot verification of four candidate proteins. **A**, peptide length. **B**, protein mass. **C**, coefficient of variation (CV). **D**, overlapping proteins. **E**, western blot verification. **F**, proteome data. **G**, western blot verification (grayscale processing). OV, ovary; IE, early intersex stage gonad; IM, middle intersex stage gonad; IL, late intersex stage gonad; TE, testis. The results are presented as the means ± SEMs. Different lowercase superscripted letters indicate significant differences (*p* < 0.05)

According to the histological changes in the gonads, the OV, IE, IM, IL, and TE stages were identified. The protein patterns in the five sexual stages were detected by iTRAQ mass spectrometry. The peptide spectrum matches (PSMs) were more than 95% credible, and each credible protein contained at least one unique peptide. Only credible PSMs and proteins were retained, and false discovery rate (FDR) verification was performed to remove peptides and proteins with FDR > 1%. The main peptide length was 4–20 amino acids (aa), and a peak was observed at 12 aa (Fig. 1A). The sizes of the enriched proteins were mainly 10–70 kDa with a peak at 20 kDa (Fig. 1B). The pros and cons of repeatability were evaluated by determining the coefficient of variance (CV) value. The CV values in the IL and IE periods were the smallest, showing good repeatability. The TE values were the largest, and the reproducibility was relatively poor (Fig. 1C).

A total of 5125 nonredundant proteins were identified through the five sexual stages (Additional file 2–3), including 4422 proteins in OV, 4430 proteins in IE, 4679 proteins in IM, 4196 proteins in IL, and 4868 proteins in TE. Only 3499 common proteins were detected in the five sexual stages (Fig. 1D). Four proteins were selected to verify the accuracy of the sequencing data by western blot (Fig. 1E-G and Additional file 1: Fig. S1*p* < 0.05), including V-type proton ATPase subunit G 1 (Vatpg1), triosephosphate isomerase (Tpi), insulin-like growth factor 2 mRNA-binding protein 3 isoform X1 (Igf2bp3), and Cu-Zn superoxide dismutase isoform X1 [(Cu-Zn) Sod1]. Among them, three proteins [Tpi, Igf2bp3, and (Cu-Zn) Sod1] had similar expression patterns in the sequencing results.

### Verification and analysis of the DEPs during sex change

**Fig. 2:**
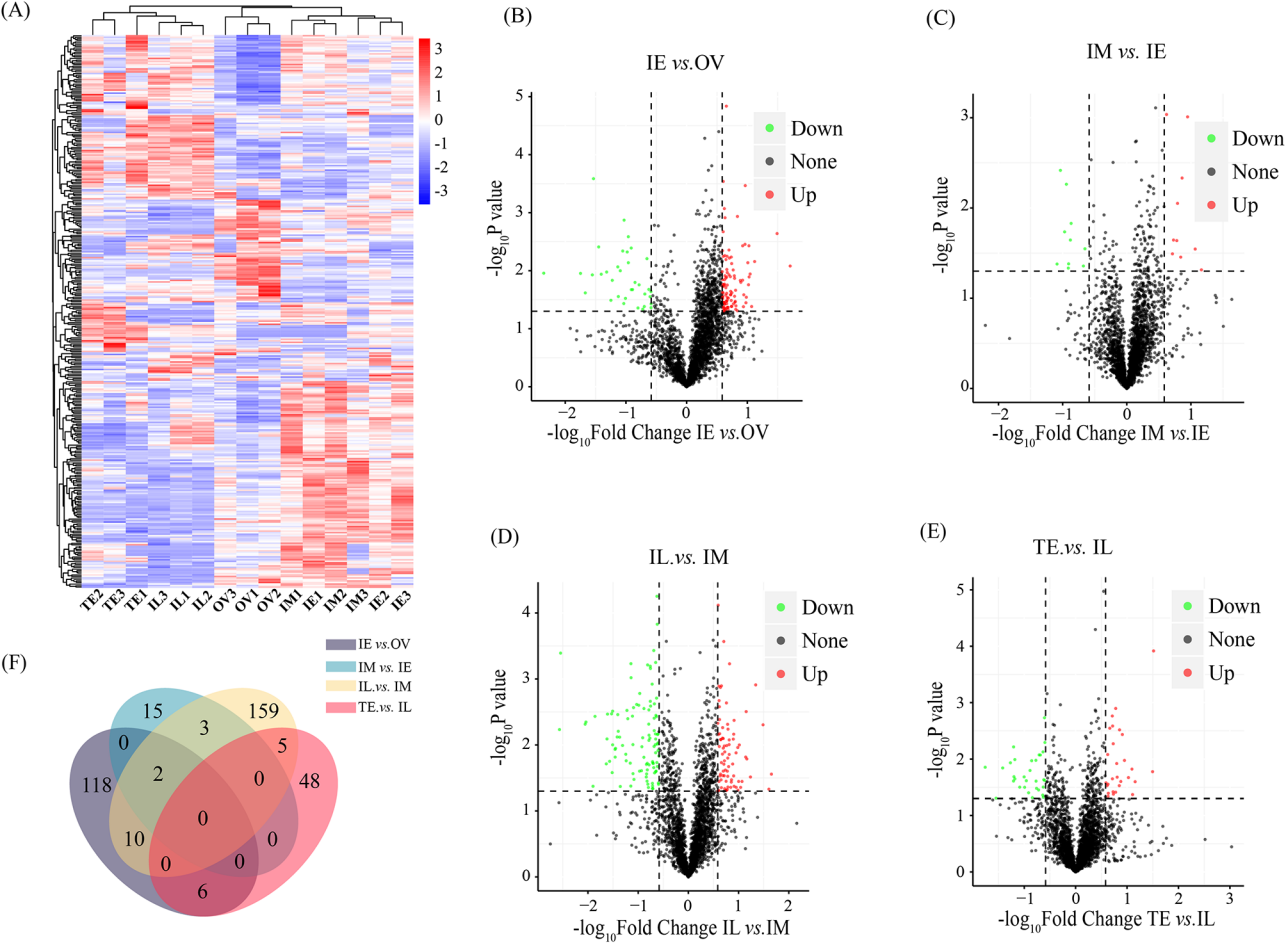
Differentially expressed proteins (DEPs) during sex change. **A**, clustering of DEGs. **B-E**, volcano maps of DEPs (B, IE vs. OV. C, IM vs. IE. D, IL vs. IM. E, TE vs. IL). **F**, overlapping DEPs. OV, ovary; IE, early intersex stage gonad; IM, middle intersex stage gonad; IL, late intersex stage gonad; TE, testis

The clustering of the DEPs showed that the five sexual stages, OV, IE, IM, IL, and TE, exhibited distinct expression patterns (Fig. 2A). A total of 394 DEPs were screened. The most DEPs were found in the IL vs. IM group comparison (101 downregulated and 78 upregulated in IL, Fig. 2D and Additional file 7), followed by the IE vs. OV groups (101 upregulated and 35 downregulated in IE, Fig. 2B and Additional file 4) and TE vs. IL groups (31 downregulated and 28 upregulated in TE, Fig. 2E and Additional file 8). Twenty DEPs were found in the IM vs. IE groups (10 downregulated and 10 upregulated in IM, Fig. 2C and Additional file 6). However, there were no common DEPs in the four comparison groups (IE vs. OV, IM vs. IE, and IL vs. IM, and TE vs. IL). Two DEPs, noelin isoform X1 and pentraxin fusion protein-like, were significantly differentially expressed in three groups (IE vs. OV, IM vs. IE, and IL vs. IM). 19 DEPs were screened in two groups, including three DEPs between the IE vs. OV and IM vs. IE groups, 10 DEPs between the IE vs. OV and IL vs. IM groups, and 6 DEPs between the IE vs. OV and TE vs. IL groups (Fig. 2F).

### Functional enrichment of total proteins

**Fig. 3:**
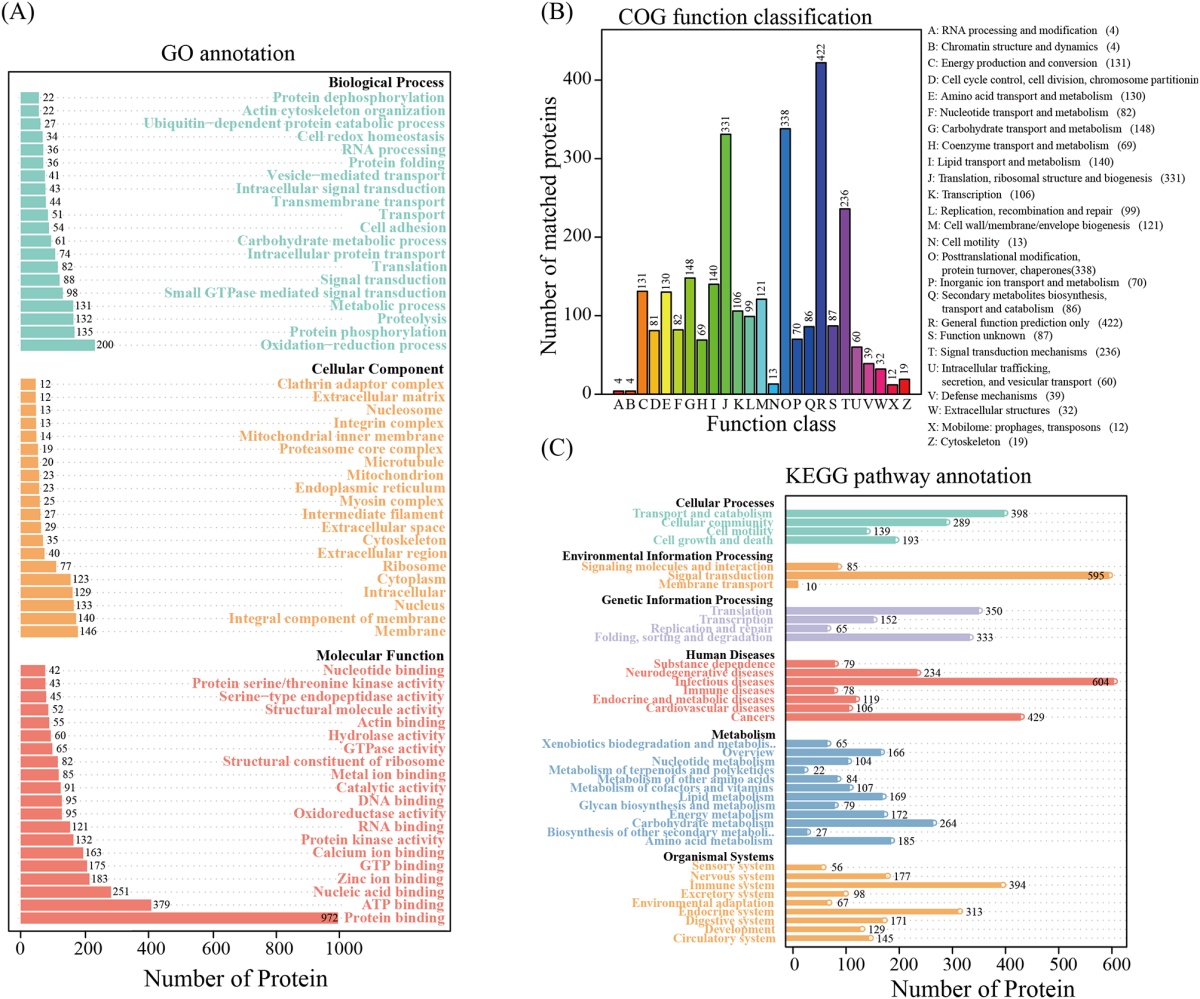
GO, COG, and KEGG analyses of proteins during sex change in the ricefield eel *Monopterus albus*. **A**, GO analysis. **B**, COG analysis. **C**, KEGG analysis. GO, Gene Ontology; COG, Clusters of Orthologous Groups; KEGG, Kyoto Encyclopedia of Genes and Genomes

The functional enrichment of the total 5125 proteins was carried out. A total of 1540 GO terms were enriched, including 699 terms in biological process, 235 terms in cellular component, and 604 terms in molecular function. The top five terms were enriched in protein binding (972 proteins), ATP binding (379 proteins), nucleic acid binding (251 proteins), oxidation‒reduction process (200 proteins), and zinc ion binding (183 proteins) (Fig. 3A and Additional file 9).

COG analysis suggested that 5125 proteins were enriched in a total of 25 classes, and the top five classes with the most abundant proteins were general function prediction only (422 proteins), posttranslational modification, protein turnover, chaperones (338 proteins), translation, ribosomal structure and biogenesis (331 proteins), signal transduction mechanisms (236 proteins), and carbohydrate transport and metabolism (148 proteins) (Fig. 3B and Additional file 10).

Furthermore, the top 10 enriched KEGG pathways were infectious diseases (604 proteins), cancers (429 proteins), signal transduction (595 proteins), transport and catabolism (398 proteins), immune system (394 proteins), translation, folding sorting and degradation (333 proteins), endocrine system (313 proteins), cellular community (289 proteins), carbohydrate metabolism (264 proteins), and neurodegenerative diseases (234 proteins) (Fig. 3C and Additional file 11).

In general, the overlapping functions from the GO, COG, and KEGG analyses were mainly concentrated in energy metabolism, signal transduction, and immune infection.

### GO analysis of sex-related DEPs

**Fig. 4:**
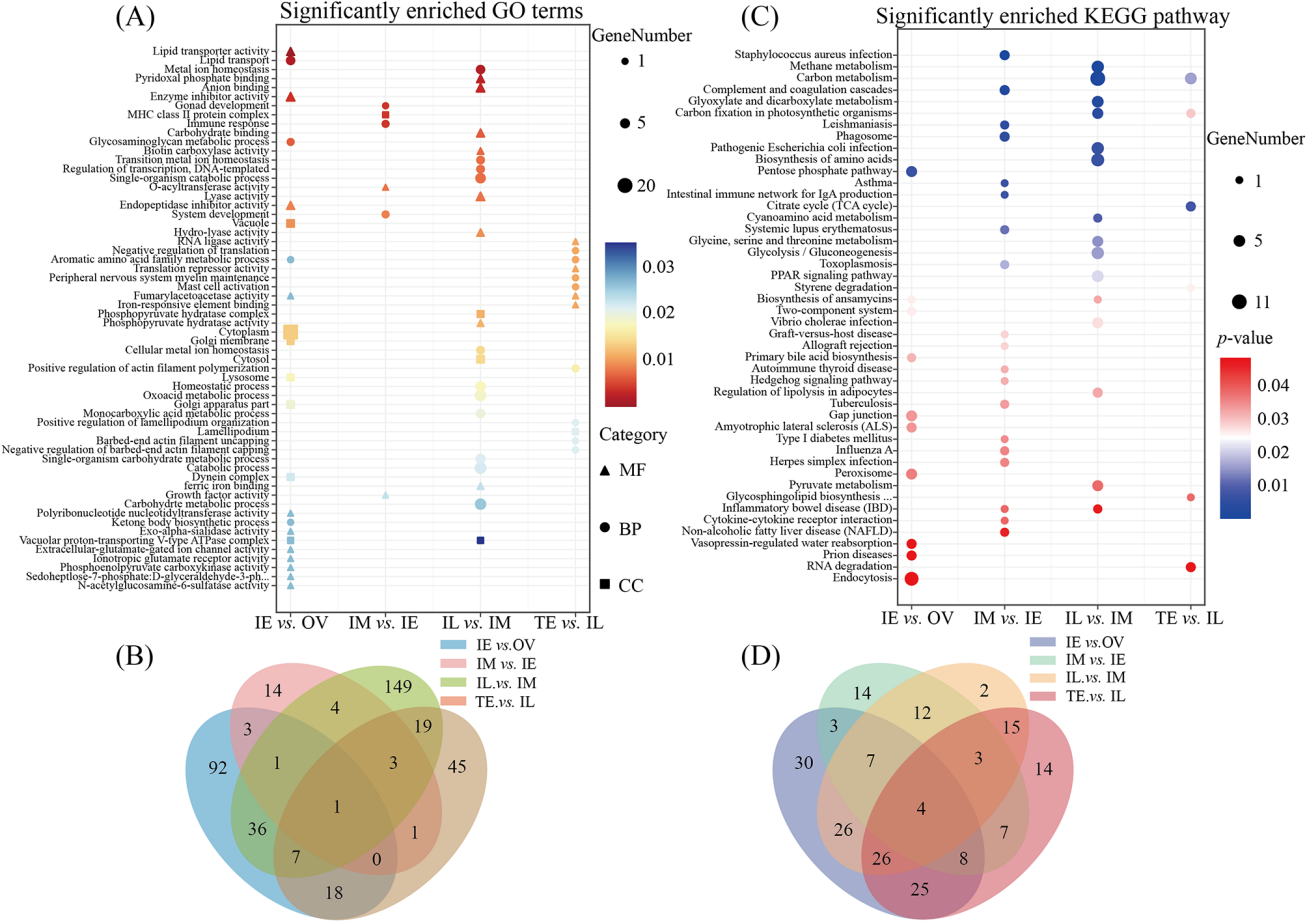
GO and KEGG analyses of differentially expressed proteins (DEPs) during sex change in the ricefield eel *Monopterus albus*. **A**, significantly enriched GO terms. **B**, overlapping terms. **C**, significantly enriched KEGG pathways. **D**, overlapping KEGG pathways. OV, ovary; IE, early intersex stage gonad; IM, middle intersex stage gonad; IL, late intersex stage gonad; TE, testis. MF, molecular function; CC, cellular component; BP, biological process

To analyze the functions of the DEPs during sex change, GO enrichment analysis was performed. The IL vs. IM group had the greatest number of GO terms (220 terms), with the most relevant terms in energy metabolism and ion metabolism, for example, ion binding, catabolic process, carbohydrate metabolic process, oxoacid metabolic process, and carboxylic acid metabolic process, etc. (Fig. 4A and Additional file 14). The IE vs. OV groups had 159 enriched terms, and the function of most the DEPs were related to posttranscriptional protein modifications, such as cytoplasm, lipid transporter activity, and lipid transport (Fig. 4A and Additional file 12). The TE vs. IL groups had 94 enriched terms, such as RNA binding, RNA processing and cell macromolecular complex assembly. Similarly, between the IL group and IM groups, there were abundant enrichments in the biological processes of ion metabolism and energy metabolism (Fig. 4A and Additional file 15). The DEPs in the IM vs. IE groups had the fewest enriched entries, and the most significant enrichment term was RNA binding (Fig. 4A and Additional file 13). Only one overlapping term was enriched in all four groups, which was immune response (Fig. 4B).

Importantly, the most abundant DEPs in the IM vs. IL groups were enriched in ion metabolism, such as ion binding, anion binding, metal ion homeostasis, and cellular metal ion homeostasis. These results suggest that ion metabolism may have important roles in the sex change process of ricefield eel. GO-Slim molecular function analysis was used to determine the functional classification. The main types of metal metabolites-related terms were molecular functions and biological processes. To further explore the potential functions of metal metabolite-related DEPs in sex change, we screened the relevant terms of four metal metabolites according to the GO database: Cu^2+^, Ca^2+^, Zn^2+^ and Fe^2+^/Fe^3+^ (Table 1). The DEPs of these metal metabolites were mainly enriched in the IM vs. IL group, with the calcium and iron terms being the most abundant. Cu^2+^ was enriched in only the IL vs. IM group, and Zn^2+^ had only one term for zinc ion binding. These data also suggest that metal metabolites are mainly related with the IM vs. IL comparison group, with an important role in male development.

**Table 1:** Metal metabolites-related terms in the ricefield eel *Monopterus albus*

Function class	GO enrichment term(s)	Term type	Comparison
Fe^2+^/Fe^3+^	Heme binding	MF	IE vs. OV
	Ferric iron binding Iron ion transport Cellular iron ion homeostasis Transition metal ion binding	MF BP BP MF	IL vs. IM
	Iron ion binding Cellular iron ion homeostasis Iron ion transport Iron-responsive element binding	MF BP BP MF	TE vs. IL
Cu^2+^	Copper ion homeostasis Copper ion binding Transition metal ion binding	BP MF MF	IL vs. IM
Zn^2+^	Zinc ion binding	MF	IM vs. IE
Ca^2+^	Neurotransmitter secretion Calmodulin binding Calcium-dependent phospholipid binding	BP MF MF	IE vs. OV
	Calcium ion binding	MF	IM vs. IE
	Calcium ion binding Cellular calcium ion homeostasis Calcium-dependent phospholipid binding	MF BP MF	IL vs. IM
	Calcium ion binding	MF	TE vs. IL

Notes: BP, biological process; MF, molecular function; OV, ovary; IE, early intersex stage gonad; IM, middle intersex stage gonad; IL, late intersex stage gonad; TE, testis.

### KEGG enrichment of sex-related DEPs

KEGG enrichment analysis was performed on the DEPs, and significant terms (*p* < 0.05) were screened (Fig. 4C). Among them, the pathways with significant enrichment were mainly enriched in the IM vs. IE groups and IL vs. IM groups, and the 10 pathways with the most significant enrichment were *Staphylococcus aureus* infection, methane metabolism, carbon metabolism, complement and coagulation cascades, glyoxylate and dicarboxylate metabolism, carbon fixation in photosynthetic organisms, leishmaniasis, phagosome, pathogenic *Escherichia coli* infection, and biosynthesis of amino acids (Additional file 16–19). In addition, four overlapping pathways were enriched in all four groups, including the PPAR signaling pathway, peroxisome, legionellosis, and adipocytokine signaling pathway (Fig. 4D).

Four metal metabolites-related pathways were detected, including the HIF-1 signaling pathway, PI3K-Akt signaling pathway, TNF signaling pathway, and MAPK signaling pathway. Copper- and zinc-related pathways included two-component systems, Alzheimer’s disease, and mineral absorption. Among them, the number of enriched calcium-related pathways was the largest. Calcium-related pathways contained the insulin signaling pathway, mTOR signaling pathway, HIF-1 signaling pathway, and p53 signaling pathway. The significantly enriched DEPs and related pathways were screened (Table 2).

**Table 2:** DEPs in metal metabolite-related pathways and those with the most significant enrichment in the ricefield eel *Monopterus albus*

Function class	DEP description	Enriched pathway	Comparison
Fe^2+^/Fe^3+^	Map2k2a↑	HIF-1 signaling pathway PI3K-Akt signaling pathway	IE vs. OV
	Map2k6↑	TNF signaling pathway MAPK signaling pathway	IM vs. IE
	Eno3↑	HIF-1 signaling pathway	IL vs. IM
	Pdk1↑	HIF-1 signaling pathway	TE vs. IL
Cu^2+^	Cytb↑	Two-component system Alzheimer’s disease	IE vs. OV
	Cytochrome C Oxidase subunit 6B1↑	Alzheimer’s disease	IM vs. IE
	Prph↑	Amyotrophic lateral sclerosis (ALS)	IL vs. IM
	Ndufb5↑	Alzheimer’s disease	TE vs. IL
Zn^2+^	Cytb↑	Two-component system Alzheimer’s disease	IE vs. OV
	Cytochrome C Oxidase subunit 6B1↑	Alzheimer’s disease	IM vs. IE
	Fth1a↓	Mineral absorption	IL vs. IM
	Ndufb5↑	Alzheimer’s disease	TE vs. IL
Ca^2+^	Ubiquitin-Conjugating enzyme E2 L3-Like↑	Parkinson’s disease Ubiquitin mediated proteolysis	IE vs. OV
	Gnpat↑	Peroxisome	IM vs. IE
	Diaph1↓	Shigellosis Regulation of actin cytoskeleton AGE-RAGE signaling pathway in diabetic complications	IL vs. IM
	F-Actin-Uncapping protein LRRC16A-Like↑	NOD-like receptor signaling pathway Pertussis	TE vs. IL

Note: ↑, upregulated; ↓, downregulated; OV, ovary; IE, early intersex stage gonad; IM, middle intersex stage gonad; IL, late intersex stage gonad; TE, testis; DEPs, differentially expressed proteins.

### Identification of sex-related DEPs during sex change

The most significant KEGG pathways and GO terms in each comparison group based on *p* < 0.05 were screened. KEGG pathways were mainly enriched in the pentose phosphate pathway, *Staphylococcus aureus* infection, methane metabolism, carbon metabolism, and the TCA cycle. The main GO terms included cytoplasm, ion binding, and RNA binding. These functional annotations have been highly correlated with energy metabolism and ion binding. Then, several sex-related DEPs in these significant pathways and among these terms were identified, including matrix metalloproteinase-9 (Mmp-9), alcohol dehydrogenase 1-like isoform X1 (Adh1-l), Vatpg1, Tpi, and Igf2bp3 (Table 3).

**Table 3:** The most significantly enriched terms and pathway sex-related DEPs in the ricefield eel *Monopterus albus*

Comparison	Up	Down	GO enrichment term/enriched pathway	Sex-related DEP(s)
IE vs. OV	101	35	Cytoplasm (20)	Eif-4e1a, Ap2a1, Hmgcl, Galns, Transaldolase, Vatpg1
			Pentose phosphate pathway (4)	Transaldolase
IM vs. IE	10	10	——	——
			Staphylococcus aureus infection (3)	Complement c3-like
IL vs. IM	78	101	Ion binding (27)	Fili2, Nucb2il, Enolase-like, Cadherin-1, Annexin a1-like, Protein s100-a1-like, Mmp-9
			Methane metabolism (6) Carbon metabolism (11)	Adh1-l, Aldoa, Enolase-like, Madem, Tpi
TE vs. IL	28	31	RNA binding (5)	Igf2bp3
			TCA cycle (3)	——

Notes: OV, ovary; IE, early intersex stage gonad; IM, middle intersex stage gonad; IL, late intersex stage gonad; TE, testis; DEPs, differentially expressed proteins.

### Characterization of metal metabolite -related DEPs during sex change

**Fig. 5:**
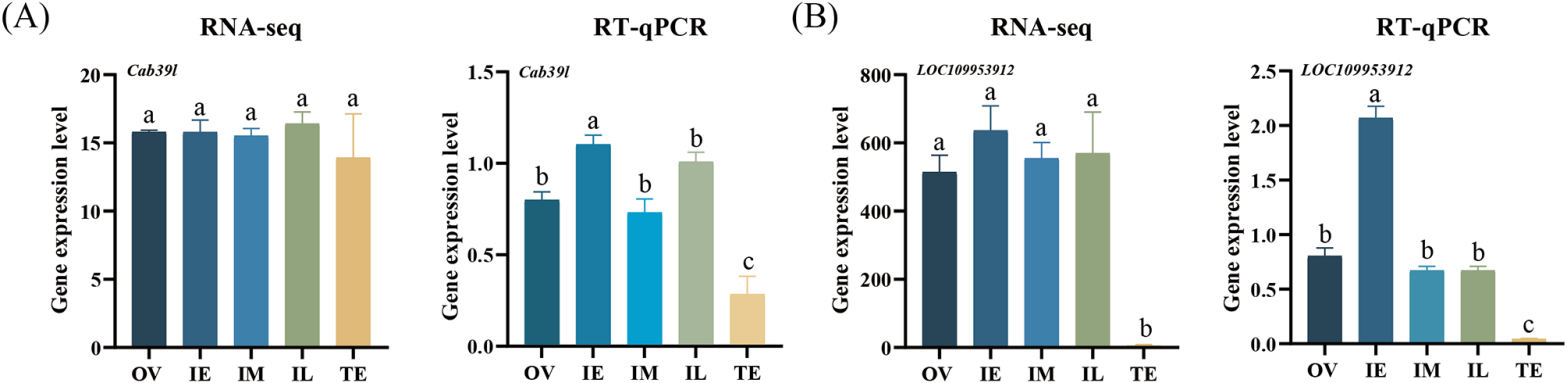
Gene expression levels of metal metabolite-related DEPs from the RNA-seq and RT‒qPCR data during sex change in the ricefield eel *Monopterus albus*. **A**, the expression level of *cab39l*. **B**, the expression level of *LOC109953912.* qRT-PCR, quantitative real-time PCR; RNA-seq, RNA sequencing; OV, ovary; IE, early intersex stage gonad; IM, middle intersex stage gonad; IL, late intersex stage gonad; TE, testis. The results are presented as the means ± SEMs. Means marked with different letters were significantly different (*p* < 0.05)

As mentioned above, a few metal metabolites-related signaling pathways related with sex changes were determined in the ricefield eel. To explore the expression patterns of these factors during sex change, a total of 111 nonredundant DEPs were identified from 15 iron-related proteins, 14 copper-related proteins, 10 zinc-related proteins, and 131 calcium-related proteins. To analyze the expression patterns of these 111 nonredundant DEPs more comprehensively, the transcriptome and proteome data from the five stages were analyzed based on the log_2_-fold change at both the protein level and mRNA level. Only four of these genes [*LOC109952670*, *LOC109953418*, *LOC109953912*, and *calcium Binding Protein 39 Like* (*cab39l*)] had consistent expression using RT‒qPCR. Two genes (*LOC109953912* and *cab39l*) matched the transcriptome data (Fig. 5 and Additional file 1: Fig. S2*p* < 0.05).

### Metal ion content during sex change

**Fig. 6:**
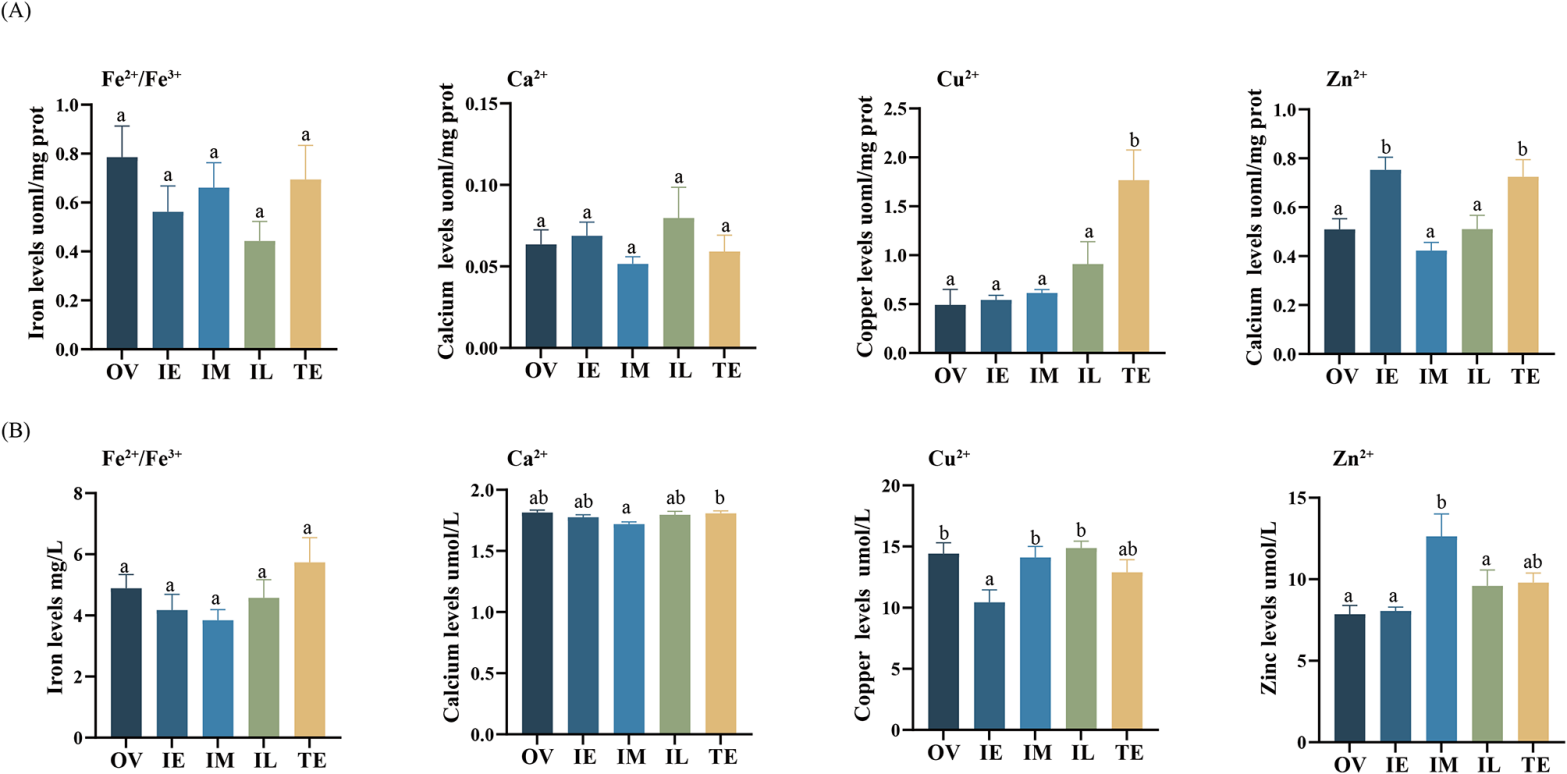
Trends in gonads and serum levels of metal metabolites during sex change in the ricefield eel *Monopterus albus*. **A**, levels of Fe^2+^/Fe^3+^, Cu^2+^, Ca^2+^ and Zn^2+^ in gonads. **B**, levels of Fe^2+^/Fe^3+^, Cu^2+^, Ca^2+^ and Zn^2+^ in serum. OV, ovary; IE, early intersex stage gonad; IM, middle intersex stage gonad; IL, late intersex stage gonad; TE, testis. Different lowercase letters indicate significant between-group differences (*p* < 0.05), while the same lowercase letters indicate that the between-group differences were not significant (*p* < 0. 05)

To investigate whether the levels of metal metabolites varied during sex change in the ricefield eel, the levels of Fe^2+^/Fe^3+^, Cu^2+^, Ca^2+^ and Zn^2+^ were measured during the five periods. The results showed that there were no significant changes in the levels of Fe^2+^/Fe^3+^ or Ca^2+^, while there were significant changes in the Cu^2+^ and Zn^2+^ levels in gonads during sex change (Fig. 6A). Importantly, the expression trends of Fe^2+^/Fe^3+^ and Ca^2+^ were opposite. In addition, the expression trend of Cu^2+^ increased with the process of sex change and reached the highest level in TE, which suggests that Cu^2+^ may be a male-biased metal metabolite. In contrast, the expression trend of Zn^2+^ increased and then decreased, and the content was the highest during the IE and TE periods. In addition, the levels of serum metal metabolites during sex change altered little for Fe^2+^/Fe^3+^ and Ca^2+^, while Cu^2+^ and Zn^2+^ remained significantly changed (Fig. 6B). However, Cu^2+^ was no longer elevated with the course of sex change, and changes in its content occurred mainly during intersex.

## Discussion

Unlike the transcriptome, the proteomics-based approach for detection the expressed proteins in tissues or cell types has complemented the genome initiatives [24, 25]. To date, there has been a gradual increase in proteomic studies in fish gonads, which have revealed changes in the protein composition of the gonads, contributing to shedding light on the gonadal status. In the gill of tropical marbled eel, salinity acclimation proteins were analyzed by iTRAQ proteomic method [26]. The significant correlation between gonadal index (GI) and protein content might play key roles in changing the condition factor of gonads in sea urchin *Strongylocentrotus nudus* [27]. The proteome of carp sperm between the immobilized and activated spermatozoa mainly involved in ubiquitin‒proteasome pathways, glycolysis, the TCA cycle, and remodeling related to sperm energy metabolism and motility [28]. Furthermore, the proteomic studies during zebrafish oocyte maturation, 190 proteins were significantly changed between immature and mature oocytes, and several proteins such as vitellogenin (Vtg3) and 14-3-3 protein involved in oocyte development [29]. Therefore, proteomics is an important tool to study fish gonad development. Two-dimensional electrophoresis was used to isolate the DEP spots in ricefield eel [22]. Over 80 DEPs were identified in ricefield eel, in which there were five spots highly expressed in the ovary, three spots in the ovotestis, 16 in the testis, 24 in both the ovary and ovotestis, and 17 in both the testis and ovotestis [22]. Chromosome group 3 (Cbx3) was highly expressed in the testis, and a member of the RAS oncogene family (Rab37) was highly expressed in the ovary of ricefield eel [22]. However, two-dimensional electrophoretic proteome analysis has limitations, in which the protein cannot be completely acquired and fully utilized in a database, and the intersex stage of research is not systematic enough for ricefield eel.

In the present study, proteins from five periods of sex change in the ricefield eel (OV, IE, IM, IL, and TE) were comprehensively and systematically investigated. There were the most DEPs from the IM period to the TE period. Especially in the IL vs. IM group, clustered heatmap male-biased DEPs were abundant. Compared to previously reported studies, our study obtained 5362 total proteins and 335 DEPs (two-dimensional electrophoresis obtained only approximately 80 proteins [22]) and performed functional enrichment analysis. In addition, we have more details on the intersex stages (IE, IM, and IL). In our previous transcriptome study, we found that apoptosis-related genes were strongly related with sex change, and we screened for *tgfb3*, *fshr*, *camk4*, and *calmodulin*, which might be key genes [30]. Although some results on the process of sex change at the transcriptional level have been obtained, the relevant studies at the protein level need to be further improved. Therefore, high-throughput proteome sequencing was utilized to characterize the expression levels of large proteins to understand the molecular mechanisms of sex change in ricefield eel.

With GO and KEGG analyses, some proteins with significant sex differences were screened, including Vatpg1, Tpi, and Igf2bp3. In mouse epididymis and vas deferens, vacuolar H^+^ ATPase was necessary for establishing an acidic lumen pH environment that kept sperm at rest during sperm maturation and storage in these organs [31, 32]. Tpi was involved in the gene expression of retinol during sperm metabolism in rats [33]. Igf2bp3 was related to embryonic development and placenta formation in medaka [34]. In our present work, Vatpg1, Tpi and Igf2bp3 were the DEPs that were markedly increased in the OV to IM period. While the gonad lamella was gradually occupied by testicular tissues from the OV to IM period, the number of ovarian tissues were gradually reduced [35]. But Vatpg1, Tpi and Igf2bp3 were significantly reduced from IM to TE stage, and then returned to level of expression similar to the OV period. The gonadal lamella was gradually filled with testicular tissues and gradual reduction of ovarian tissues until it disappeared from the IM to TE period [35]. Above results suggest that Vatpg1, Tpi and Igf2bp3 might play important roles in oocyte disappearance and spermatogenesis during sex change of ricefield eel.

Previous studies have shown that metal metabolites, such as cadmium (Cd) [36], iron (Fe) [37, 38], copper (Cu) [39], lead (Pb) [40], and zinc (Zn) [41], play key roles in gonadal development, germ cell growth and maturation, and reproduction in mammals and fish. In our study, functional enrichment analysis of the DEPs focused on metal metabolite-related pathways and terms, such as Cu^2+^-, Ca^2+^-, Zn^2+^- and Fe^2+^/Fe^3+^-related pathways and terms. Among them, the abundance of calcium-related pathways and terms was the highest. In different vertebrate lineages, the calcium and redox pathways regulate sex determination, acting as the crucial missing link between sex and the environment [42]. Moreover, two metal metabolite-related DEPs were identified (*LOC109953912* and *cab39l*) that were consistent at both transcriptomic and proteomic expression levels. These two DEPs (*LOC109953912* and *cab39l*) were enriched in the Parkinson’s disease, ubiquitin-mediated proteolysis, gap junction, and phagosome pathogenic Escherichia coli infection pathways. These results suggest that metal metabolites may be involved in several biological functions during sex change in ricefield eel.

Chronic exposure to these metal metabolites impaired mammalian follicle production and fish ovarian development by interfering with reproductive hormones and reactive oxygen species (ROS) production, which in turn damaged various molecules, including proteins, lipids, and DNA, and disrupted antioxidant defenses, ion homeostasis and endoplasmic reticulum homeostasis [40, 43, 44]. Four metal metabolites (Cu^2+^, Ca^2+^, Zn^2+^ and Fe^2+^/Fe^3+^) were detected in our study. Among them, Ca^2+^ and Fe^2+^ did not vary greatly, but there was some correlation and antagonism between their levels because Ca^2+^ affects the absorption of Fe^2+^/Fe^3+^ [45]. In contrast, the content of Cu^2+^ tended to increase gradually during sex change in our study, suggesting that Cu^2+^ might be an important metal metabolite in spermatheca development. A previous study found that Cu might cause a decrease in antioxidant capacity and testicular spermatogenesis [38]. In addition, Cu exposure upregulated the expression of oxidative phosphorylation pathway genes (*Cytochrome c oxidase*, *Sod1*, and *Gst*) in the testis to induce ROS production, which was related with Cu toxicity in the testis [38, 46, 47]. Long-term exposure to high levels of Cu had a toxic effect on yellow catfish (*Pelteobagrus fulvidraco*), whereas at low doses and after relatively short-term exposures, serum steroid hormones and steroidogenesis-related gene levels were elevated [48]. These results reflect the disruption of the balance between antioxidants and oxidants after Cu exposure, affecting ovary and testis development. In contrast, the content of Zn^2+^ showed a trend of increasing, then decreasing, and then increasing again, and was the highest in the IE period, indicating that zinc ions have an important role in the process from OV to IM. Zn is an essential nutrient involved in many physiological processes, such as follicular development, immune response, homeostasis, oxidative stress, cell cycle progression, DNA replication, DNA damage repair, apoptosis, and aging [41]. Zn deficiency could lead to blocking the oocyte meiotic process, cumulus expansion, and follicle ovulation [41]. In flounder (*Pseudopleuronectes americanus*), females stored Zn in summer for winter gonadal development [49]. Thus, metal metabolites play important functions in regulating gonadal development by influencing the balance between antioxidants and oxidants, as well as in steroid hormone production.

(Cu-Zn) Sod1 and Mmp-9 were identified as potential proteins related with crosstalk between metal metabolites and sex-related proteins in ricefield eel. (Cu-Zn) Sod1 is an abundant copper- and zinc-containing protein [50]. Its primary function is to act as an antioxidant enzyme, lowering the steady-state concentration of superoxide, but when mutated, it can also cause disease [50]. In our data, (Cu-Zn) Sod1 was mainly enriched in the peroxisome pathway. Peroxisomes are highly dynamic cell organelles that play key roles in cellular lipid and hydrogen peroxide (H_2_O_2_) metabolism [51]. In the present work, the abundances of (Cu-Zn) Sod1 in the intersexual stages (especially during the IM stage) were significantly higher than those in the OV and TE stages, suggesting that (Cu-Zn) Sod1 had been associated with the sex change process in the ricefield eel. The expression level of Mmp-9 was progressively lower with sex change, suggesting that it is likely to be a male-related protein. Mmp-9 has been immunolocalized during the development of the follicular membrane and interstitium in rodents [52]. The level of Mmp-9 in sheep follicles was elevated from normal follicles to atresia follicles [53]. High expression levels of Mmp-9 were also accompanied by an increase in the rate of follicular apoptosis in patients [54]. These data suggest that (Cu-Zn) Sod1 and Mmp-9 are likely to influence gonadal development during sex change by affecting the level of ROS. In the future, we may perform more detailed functional studies on (Cu-Zn) Sod1 and Mmp-9 in the sex change process of ricefield eel.

## Conclusion

In summary, we analyzed the protein profiles of ricefield eel gonadal tissues in five stages (OV, IE, IM, IL, and TE) and verified the plausibility of the data using western blotting. Then, we analyzed the functional enrichment of metal metabolite-related DEPs. Sex-related DEPs (Vatpg1, Tpi and Igf2bp3) may play important roles in oocyte disappearance and spermatogenesis during sex change. In addition, we detected the contents of the metal ions Zn^2+^, Cu^2+^, Ca^2+^, and Fe^2+^/Fe^3+^ at these five stages and screened for (Cu-Zn) Sod1 and Mmp-9 as possible key proteins in the sex reversal process. Our data therefore nicely complement the protein data from ricefield eel. As mentioned above, through proteome analysis of the sex change stage of the ricefield eel, detailed genetic information about the mechanism of sex change has been more deeply described, including ovarian maturation and degeneration, gonadal differentiation, and sex changes.

## Methods

### Experimental animals

**Fig. 7:**
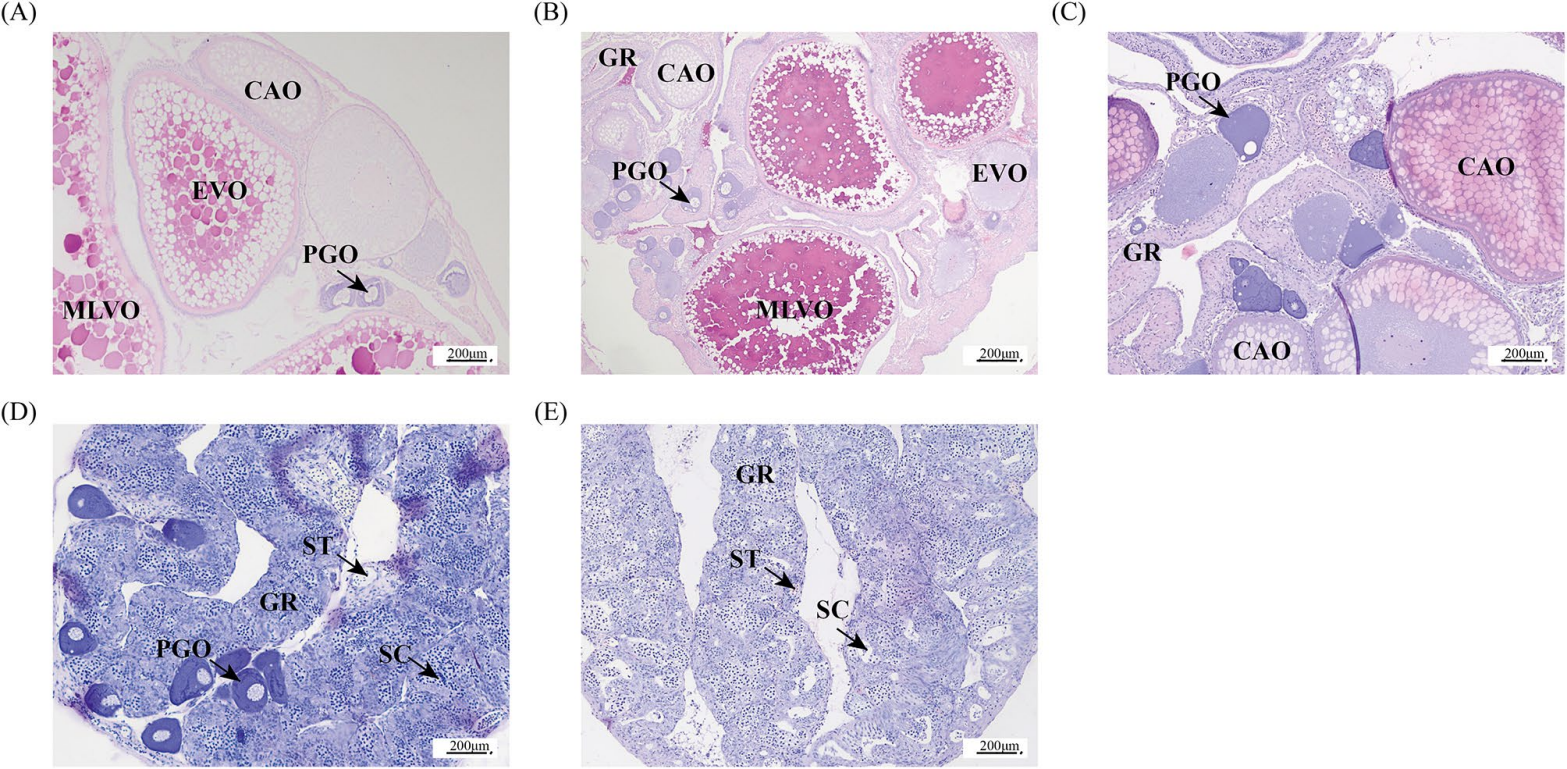
Identification of the gonad developmental stages in the ricefield eel *Monopterus albus*. **A**, ovary (OV). B, early intersex gonad (IE). **C**, middle intersexual gonad (IM). **D**, late intersex gonad (IL). **E**, testis (TE). CAO, cortical alveolar oocyte; PGO, primary growth stage oocyte; EVO, early vitellogenesis oocyte; GR, gonadal ridge; SC, spermatocyte; ST, spermatid

Wild ricefield eels (*n* = 210, body length = 39.42 ± 5.13 cm, and body weight = 48.53 ± 24.67 g) were purchased from a local market (Chengdu, Sichuan, China). The fish were maintained in the laboratory at a water temperature of 21.7 ± 2.5 °C under a photoperiod of 16 h light:8 h dark. Fish were decapitated after anesthesia with 0.02% tricaine buffer (80 µg/L) (Sigma, Saint Louis, MO, USA). Then, the gonads were collected and immediately stored in liquid nitrogen at − 80 °C. The gonads were divided into two parts. The first was fixed with Bouin’s solution for 24 h and then stored in 75% ethanol for determination of the gonadal developmental stage, while the second was immediately stored in liquid nitrogen at − 80 °C until RNA and protein extraction. Histological classification of the gonads, including OV, IE, IM, IL, and TE, has been described previously [16]. Three biological replicates were randomly selected from gonadal tissues at the OV, IE, IM, IL, and TE stages for proteome sequencing (Fig. 7).

### Total protein extraction, isobaric tags for relative and absolute quantification (iTRAQ) peptide labeling, and liquid chromatography–tandem mass spectrometry (LC‒MS/MS) analysis

Each sample was ground individually in liquid nitrogen and lysed with PASP lysis buffer (100 mM NH_4_HCO_3_, 8 M urea, pH 8), followed by 5 min of ultrasonication on ice. The lysate was centrifuged at 12,000 × g for 15 min at 4 °C, and the supernatant was reduced with 10 mM DL-dithiothreitol for 1 h at 56 °C and subsequently alkylated with sufficient iodoacetamide for 1 h at room temperature in the dark. Then, each sample was completely mixed with 4 volumes of precooled acetone by vortexing and incubated at -20 °C for at least 2 h. The samples were then centrifuged at 12,000 × g for 15 min at 4 °C, and the precipitate was collected. After washing with 1 mL of cold acetone, the pellet was dissolved in dissolution buffer.

The volume of each protein sample was made up to 100 µL with DB dissolution buffer (8 M urea, 100 mM triethylammonium formate, pH8.5). Trypsin and 100 mM triethylammonium formate buffer were added, and the sample was digested at 37 °C for 4 h. Then, trypsin and CaCl_2_ were added, and the sample was digested overnight. Formic acid was mixed with the digested sample, which was adjusted to pH 3 and centrifuged at 12,000 × g for 5 min at room temperature. The supernatant was slowly loaded onto a C18 desalting column, which was washed with washing buffer (0.1% formic acid, 3% acetonitrile) 3 times, and then the sample was eluted with elution buffer (0.1% formic acid, 70% acetonitrile). The eluents of each sample were collected and lyophilized. Twenty microliters of 1 M TEAB buffer was added for reconstitution, and enough iTRAQ labeling reagent (dissolved in isopropanol) was added. The sample was mixed with shaking for 2 h at room temperature. Then, the reaction was stopped by adding 100 µL of 50 mM Tris-HCl (pH = 8). Equal volumes of each labeled sample were combined, mixed, desalted, and lyophilized. For multiple labeled groups, a common reference was created by pooling an equal quantity of each sample.

Samples were injected into homemade C18 Nano-well columns. The peptides were isolated using the linear gradients listed for the homemade analytical columns. The isolated peptides were analyzed by Q Exactive™ series mass spectrometers (Thermo Fisher) with a Nanosury Flex™ ion source (ESI), a spray voltage of 2.1 kV, and an ion transport capillary temperature of 320 °C. The following parameters were used: full scan range from m/z 407 to 1500, a resolution of 60,000 (at m/z 200), an automatic gain control target of 3 × 10^6^, and a maximum ion implantation time of 20 ms. The top 40 precursors with the highest abundance in the full scan were selected for fragmentation by high-energy collision dissociation and analyzed by MS/MS at a resolution of 15,000 (at m/z 200), an automatic gain control target value of 5 × 10^4^, a maximum ion implantation time of 45 ms, a normalized collision energy of 32%, an intensity threshold of 2.2 × 10^4^, and a dynamic rejection parameter of 20 s.

### Analysis of the DEPs

The relative quantitative values of the unique peptides were obtained based on the peak area from the original spectrum and peptide spectrum matches (PSMs) by Proteome Discoverer 2.2 (PD 2.2, Thermo). The relative quantitative value of each unique peptide could be corrected based on the quantitative information from all unique peptides in each protein. The protein quantitation results were statistically analyzed by T test, and the corresponding *p* value was calculated as a significance indicator. When FC ≥ 1.5 and *p* ≤ 0.05, the protein was upregulated, and when FC ≤ 0.67 and *p* ≤ 0.05, the protein was downregulated.

Gene Ontology (GO) functional analysis was conducted by the program InterProScan against nonredundant protein databases (including Pfam, PRINTS, ProDom, SMART, ProSite, and PANTHER) [55]. Then, the Clusters of Orthologous Groups (COG) and Kyoto Encyclopedia of Genes and Genomes (KEGG) databases were utilized to identify the protein families and pathways. Volcano maps, cluster heatmaps, and GO, COG and KEGG enrichment analyses of the DEPs are presented [56].

### Joint transcriptome-proteome analysis

Correlation analysis of the transcriptome and proteome data at the fold change of the two omics was carried out. Some metal-related genes/proteins from the transcriptome and proteome data were screened. Then, the expression patterns of these coidentified genes/proteins based on the log_2_-fold change values were analyzed. A log_2_-fold change > 0 indicated upregulation, and a log_2_-fold change < 0 indicated downregulation.

### Western blot verification

Total protein was extracted from the ricefield eel gonads by the Tissue or Cell Total Protein Extraction Kit (Sangon Biotech C510003). All components were loaded with equal amounts of protein extract on a 12% polyacrylamide gel (standard gel, Bio-Rad), and the isolated proteins were transferred to a polyvinylidene fluoride membrane. The primary antibodies included those against the following proteins: Igf2bp3 polyclonal antibody (1:8000, Proteintech, 14642-1-AP, 86% homology), Tpi rabbit polyclonal antibody (1:4000, Proteintech, 10713-1-AP, 89% homology), Atp6v1g1 rabbit polyclonal antibody (1:2000, Proteintech, 16143-1-AP, 84% homology), and Sod1 rabbit pAb (1:1000, ABclonal, A0274, 70% homology). According to the molecular weight of proteins, specific bands for Igf2bp3 (64 kDa), Tpi (27 kDa), Vatpg1 (14 kDa) and Sod1 (16 kDa) were first detected in the gonad tissue of ricefield eel (Additional file 1: Fig S3). Therefore, membranes are cropped on the position of these proteins at scale of 10–20 kDa above and below and then the antibody incubations were performed. The protein concentration was determined by BCA (Beyotime, P0012S), and the band values were quantified by ImageJ software.

### Quantitative real-time PCR (qRT‒PCR) verification

Total RNA was isolated from gonads in different developmental stages using TRIzol reagent (Invitrogen, Chicago, IL, USA) and then reverse-transcribed into cDNA using a RevertAid First Strand cDNA Synthesis Kit (Thermo Scientific, Waltham, MA, USA) according to the manufacturer’s protocol. qRT‒PCR was performed using a CFX Connect system (Bio-Rad, Chicago, IL, USA) in a final reaction volume of 10 µL comprising 5 µL of 2× SYBR Green Master Mix (TaKaRa, Dalian, China), 0.4 µL of each primer (10 µmol/L), 3.2 µL of nuclease-free water, and 1 µL of cDNA template. The cycling parameters were 95 °C for 5 min followed by 40 amplification cycles of 95 °C for 10 s, 59 °C for 15 s, and 72 °C for 20 s. The specificity of PCR amplification was confirmed by melting curve analysis, agarose gel electrophoresis, and sequencing of the PCR products. To minimize variation due to differences in cDNA loading, the expression levels of the target genes were normalized to the geometric mean expression levels of *elongation factor-1 alpha* (*ef1α*) [57]. 2^-^^Δ^^Δ^^CT^ was performed to identify the mRNA gene expression levels according to the cycle threshold values [58]. Differential expression levels were compared by the IBM SPSS Statistics 20 system at a significance level of less than 0.05 [59].

The mRNA levels of *LOC109952670*, *LOC109953418*, *LOC109953912*, and *cab39l* were normalized to the geometric mean expression levels of *ef1α*. The sequences of all the qRT‒PCR primers used in this study are provided in Table 4.

**Table 4:** Primers for real-time quantitative PCR analysis

Primer	Sequence (5′-3′)
*ef1α F*	CGCTGCTGTTTCCTTCGTCC
*ef1α R*	TTGCGTTCAATCTTCCATCCC
*LOC109952670 F*	GTTCTGGCTACAAAGGAGCG
*LOC109952670 R*	AGCCTTGCACTTGATGACCT
*LOC109953418 F*	AGCTGACAGTCTGTAGGAATGATG
*LOC109953418 R*	TGGTGGATGTGCTCTCACAA
*LOC109953912 F*	TCACCATACAGCGAGGTGAC
*LOC109953912 R*	TTCCTCCAGGTACGTCATGC
*cab39l F*	ATCATGCCGCTGTTCGGTAA
*cab39l R*	GCCACTGTTGTACAGCTCCT

Notes: F, forward; R, reverse.

### Metal and enzyme activity assays

The contents of Zn^2+^ (E011-1-1), Cu^2+^ (E010-1-1), Ca^2+^ (C004-2-1), and Fe^2+^/Fe^3+^ (A039-2-1) were determined by their respective detection kits from Nanjing Jiancheng Bioengineering Institute. All procedures were performed according to the instructions.

### Statistical analysis

The results are shown as the mean ± standard error (SE) and were analyzed by GraphPad Prism 8.0 software. Technical and biological triplicates were performed for each experiment. The differences in experimental data among groups were analyzed by one-way analysis of variance (ANOVA). A value of *p* < 0.05 was considered to indicate statistical significance.

### Electronic supplementary material

Below is the link to the electronic supplementary material.

Supplementary Material 1

Supplementary Material 2

Supplementary Material 3

Supplementary Material 4

Supplementary Material 5

Supplementary Material 6

Supplementary Material 7

Supplementary Material 8

Supplementary Material 9

Supplementary Material 10

Supplementary Material 11

Supplementary Material 12

Supplementary Material 13

Supplementary Material 14

Supplementary Material 15

Supplementary Material 16

Supplementary Material 17

Supplementary Material 18

## Data Availability

The datasets generated and analysed during the current study are available in the iProX database with accession number IPX0007324000 (https://www.iprox.cn//page/SCV017.html?query=IPX0007324000). The datasets analysed during this study are included in this published article and its supplementary information files. Please contact Zhi He (zhihe@sicau.edu.cn) if someone wants to request the data from this study.
