# Supplementary material for: Protein expression patterns and metal metabolites in a protogynous hermaphrodite fish, the ricefield eel (Monopterus albus)
Source: BMC Genomics. 2024 May 21;25:500. doi: 10.1186/s12864-024-10397-w (PMC11106920; doi:10.1186/s12864-024-10397-w)
Supplement: Supplementary file 18 — Supplementary Material 18 [file 12864_2024_10397_MOESM18_ESM.pdf]

(A)

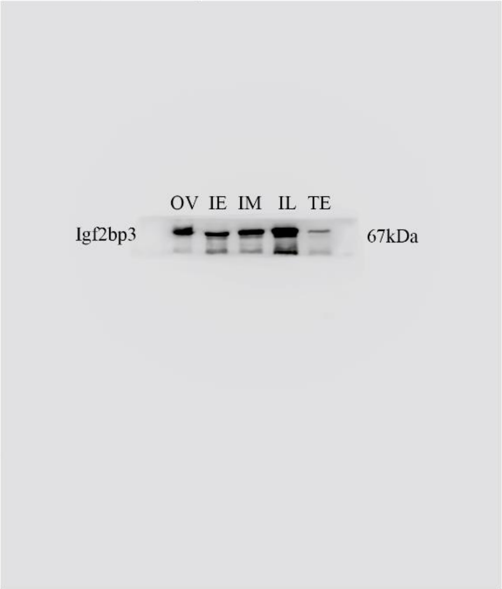
OV IE IM IL TE
Igf2bp3
67kDa

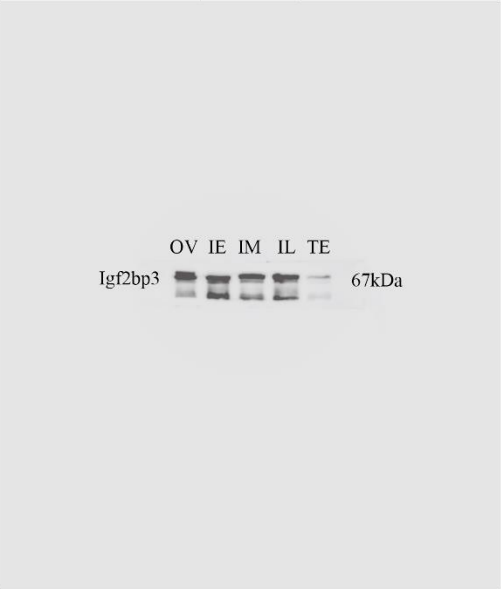
OV IE IM IL TE
Igf2bp3
67kDa

(B)

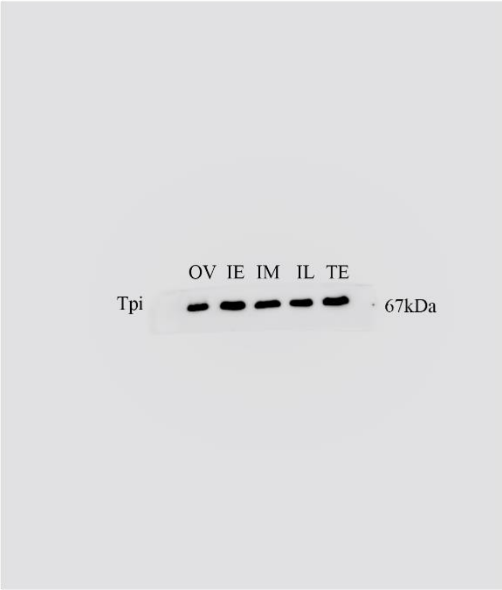
OV IE IM IL TE
Tpi
67kDa

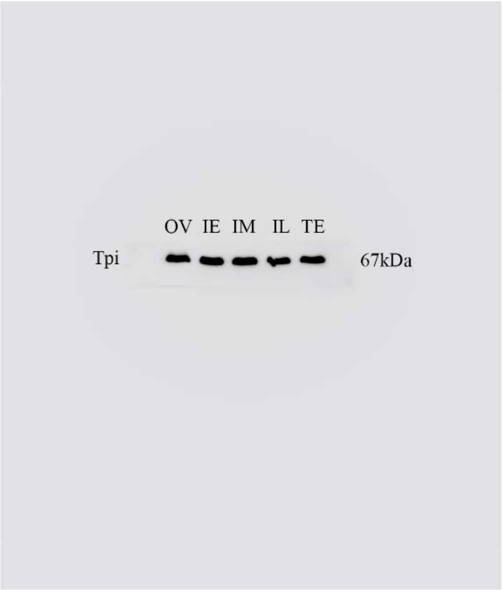
OV IE IM IL TE
Tpi
67kDa

(C)

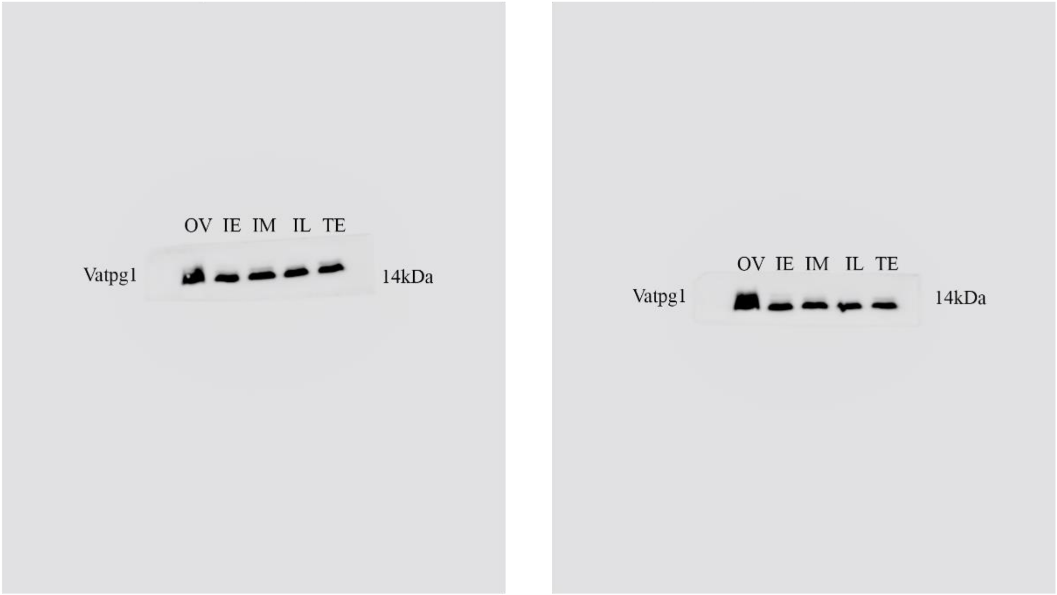

(D)

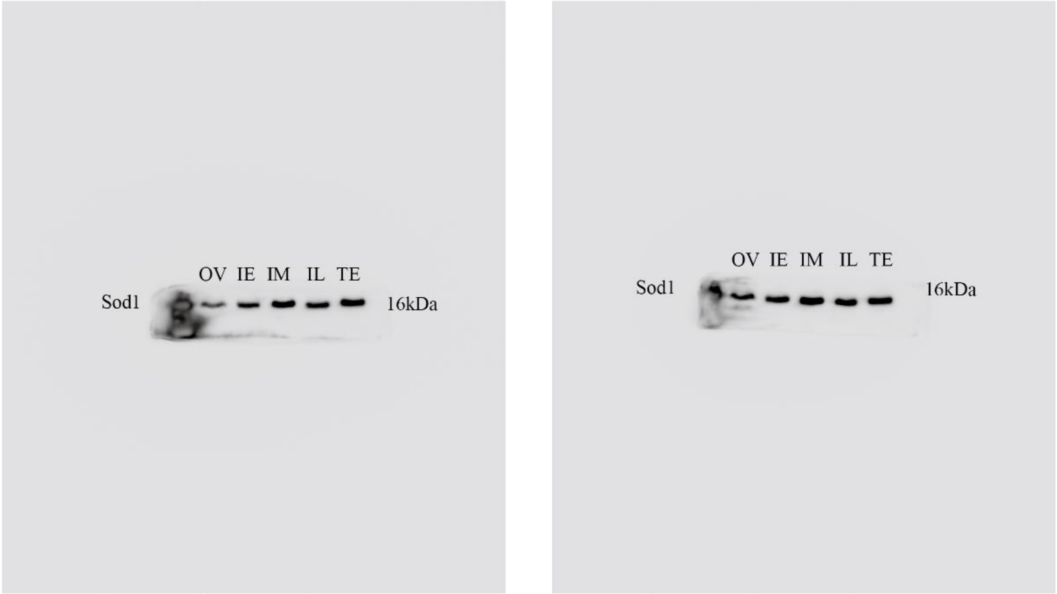

Fig. S1 The unprocessed image of western blots of Igf2bp3, Tpi, Vatpg1 and Sod1 in the ovary (OV), early intersexual stage gonad (IE), middle intersexual stage gonad (IM), late intersexual stage gonad (IL), and testis (TE) samples. **A** Igf2bp3 protein. **B** Tpi protein. **C** Vatpg1 protein. **D** Sod1 protein.

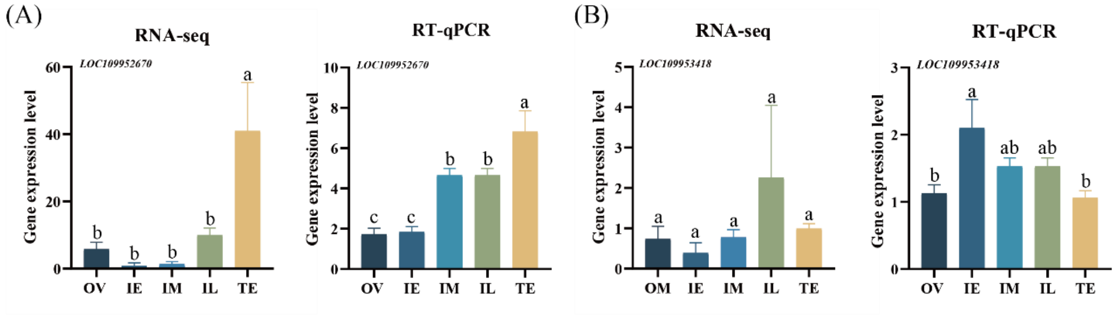

Fig. S2 Gene expression levels of metal metabolite-related DEPs from the RNA-seq and RT–qPCR data during sex change in the ricefield eel *Monopterus*. **A**, *LOC109952670*. **B**, *LOC109953418.* qRT–PCR, quantitative real-time PCR; RNA-seq, transcriptome sequencing; OV, ovary; IE, early intersex stage gonad; IM, middle intersex stage gonad; IL, late intersex stage gonad; TE, testis. The results are presented as the means ± SEMs. Means marked with different letters were significantly different ($p < 0.05$).

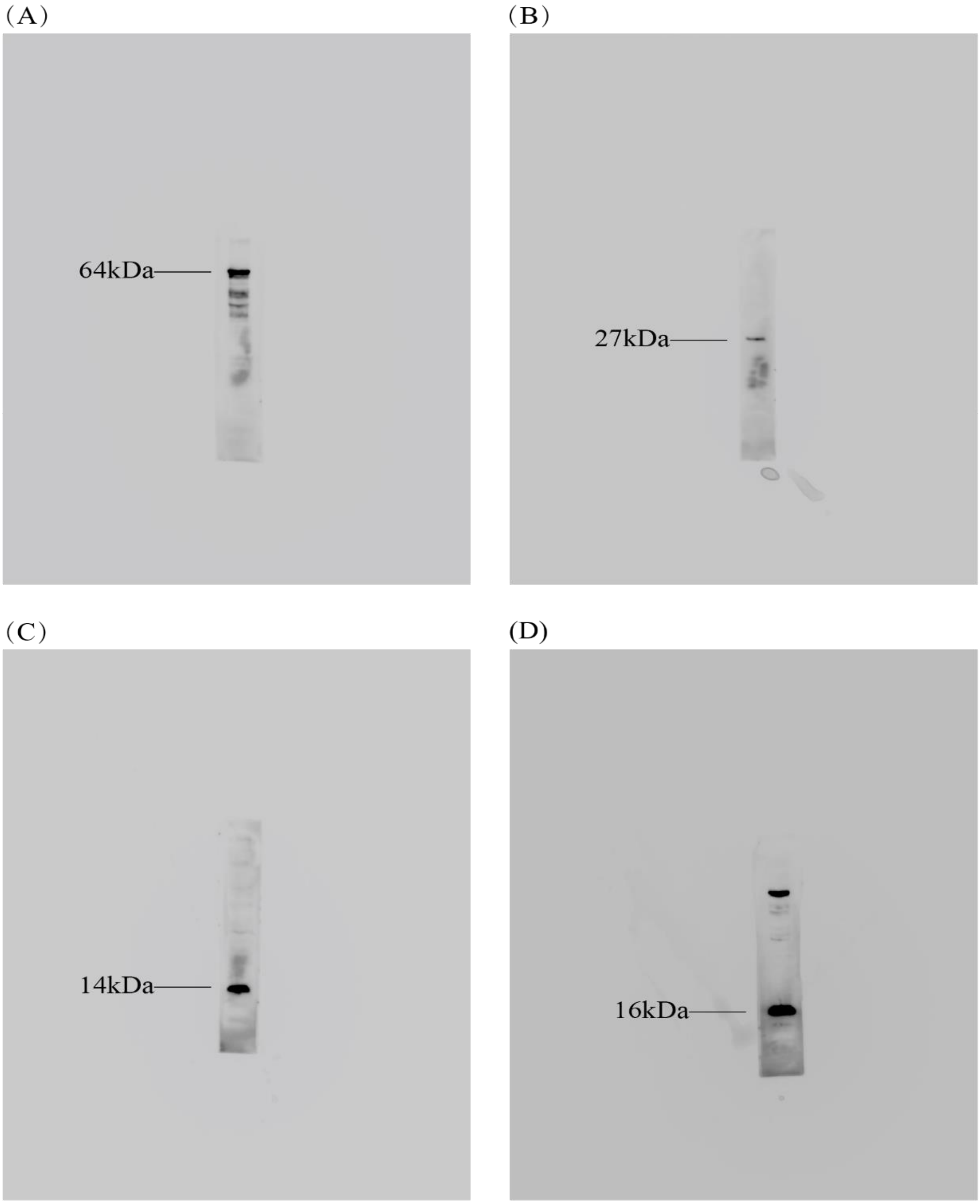

Fig. S3 Location of western blot for Igf2bp3, Tpi, Vatpg1 and Sod1 in gonadal tissues of ricefield eel *Monopterus*. **A**, Igf2bp3 at 64 kDa; **B**, Tpi at 27 kDa; **C**, Vatpg1 at 14 kDa; **D**, Sod1 at 16kDa.
